# Pulmonary Adenocarcinoma Presenting as Paraspinal Muscle Metastatic Mass

**DOI:** 10.1155/2018/5719382

**Published:** 2018-07-19

**Authors:** Matthew Harrison, Amanda Jones, Abebe Abebe

**Affiliations:** ^1^Department of Internal Medicine, Internal Medicine Residency, University of Kansas Medical Center, 3901 Rainbow Blvd. MS 1020, Kansas City, KS 66160, USA; ^2^Department of Internal Medicine, Division of General and Geriatric Medicine, University of Kansas Health System, 4000 Cambridge MS 1020, Kansas City, KS 66160, USA

## Abstract

A 39-year-old male presented with a painful paraspinal mass, which had been present for several weeks. The mass had previously been treated with oral sulfamethoxazole and trimethoprim DS, as the patient reported a history of “boils,” with no improvement in his pain or size of the mass. No further diagnostic workup was pursued until he was admitted with intractable pain. Eventual biopsy revealed adenocarcinoma, likely of pulmonary origin. This report, as well as other incoming cases, highlights this rare phenomenon of muscular metastases as the sole presentation of a distant primary malignancy.

## 1. Introduction

Skeletal muscle metastases are very uncommon with varying reported incidence rates from 0.8 to 1.6% derived from autopsy series [1, 2]. The most frequent presentation of muscular metastasis is pain with or without swelling [2]. We report an unusual case of muscular metastases as the sole presentation of a distant primary malignancy.

## 2. Case Report

**Figure 1:**
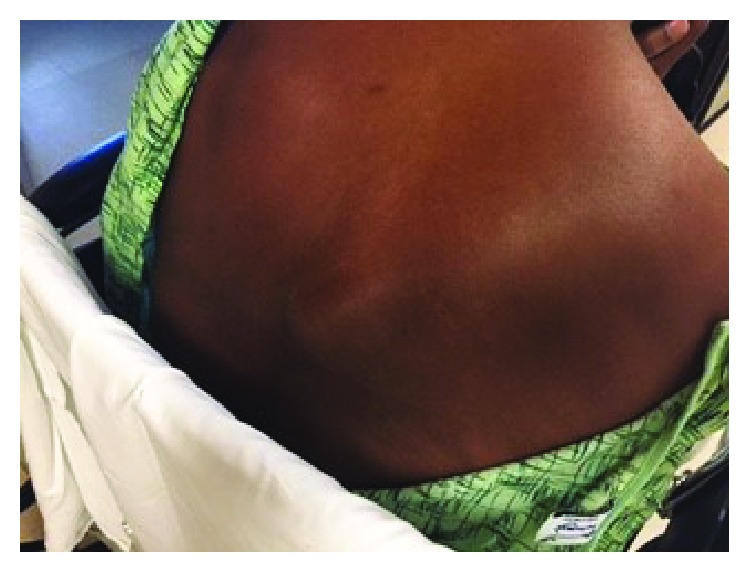
Initial clinical presentation of mass.

**Figure 2:**
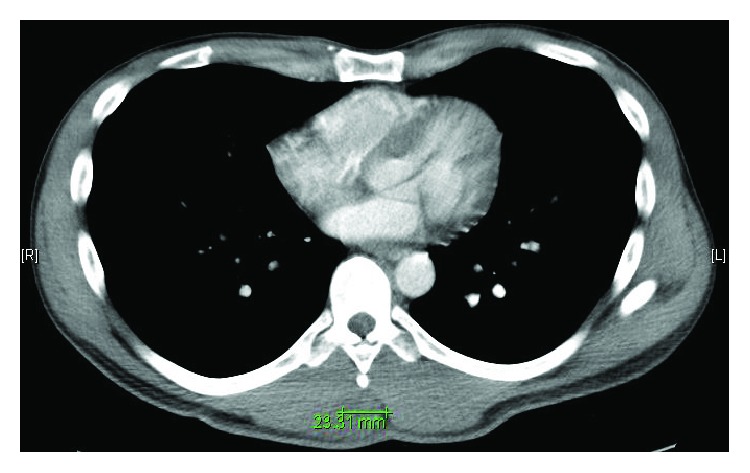
Mass noted on CT imaging.

**Figure 3:**
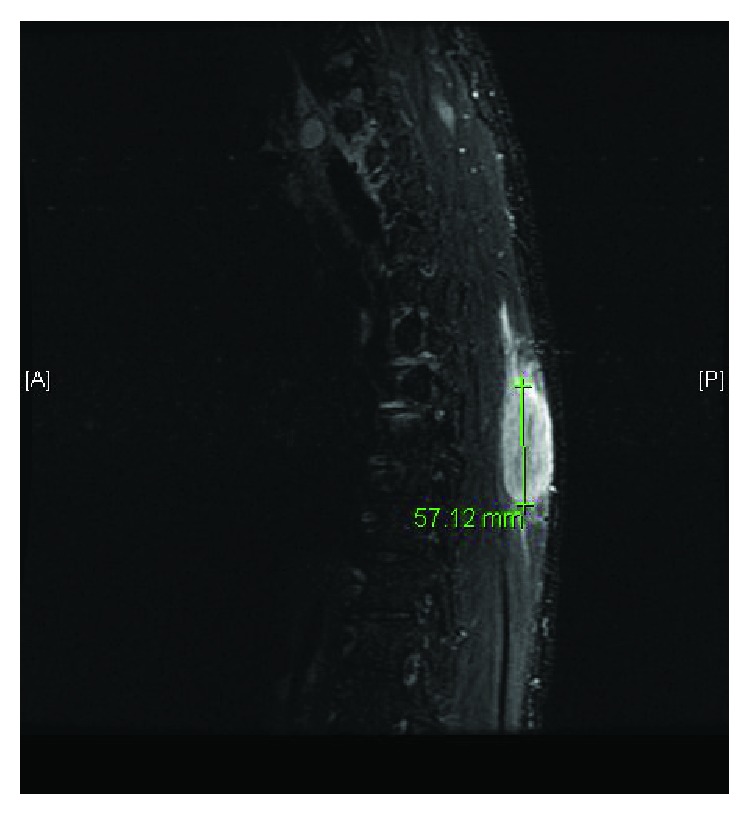
Intramuscular mass well demarcated with MRI imaging.

A 39-year-old male presented with a one-week history of a progressive, painful right paraspinal mass. He reported a history of subcutaneous abscesses which were typically treated with oral antibiotics. His current mass progressed in size and became exquisitely painful despite a recent trial of outpatient Bactrim (sulfamethoxazole and trimethoprim) DS. Examination revealed a firm, tender, nonfluctuant, and nonmobile right-sided paraspinal mass with mild erythema and without drainage (Figure 1). Slight ptosis of his right eye and intermittent right arm numbness were also noted. His laboratory data demonstrated no evidence of infection with a white blood cell count of 5.9 k/*μ*l without bandemia. Remaining complete blood count values included hemoglobin of 17.5 gm/dl and a platelet count of 441 k/*μ*l. A chemistry panel was notable for a bicarbonate of 33 mmol/l and a creatinine of 1.31 mg/dl. Computed tomography described a 2.7 × 3.3 cm mass involving the right inferior trapezius muscle without gas or fluid collections as well as a 3.9 cm right apical lung lesion (Figure 2). An MRI of the T-spine showed the initial mass with additional smaller masses in the paraspinous musculature (Figure 3). Percutaneous biopsy was consistent with metastatic adenocarcinoma of unknown primary, likely from GI or pulmonary source. Staging PET revealed hypermetabolic right apical lung mass and paratracheal nodes, as well as hepatic, left adrenal, and paraspinous muscle masses. The patient received the first 5 of 10 fractions of radiation therapy during his initial admission and was discharged with outpatient oncology and radiation oncology follow-up.

## 3. Discussion

Lung cancers are diagnosed at a metastatic stage in 40–50% of cases [1]. Skeletal muscle metastases are uncommon and usually discovered at autopsy with studies suggesting an incidence as low as 0.8–1.6% [2, 3]. This metastatic rarity is thought to be because of the unfavorable environment for tumor cells due to the muscle contraction, lactic acid production, low pH, variability of blood flow, and strong immune response of skeletal muscles [4].

Nearly all types of lung carcinoma, along with renal, bladder, and gastrointestinal tract carcinomas, have been known to metastasize to skeletal muscle [3, 5, 6]. The most common metastatic sites of lung cancer are the adrenal glands, liver, bone, and brain—as seen with the renal and liver lesions discovered in our case [7]. Frequent sites of skeletal muscle metastatic involvement, specifically in lung cancer, include the thigh, iliopsoas, and paraspinous muscles, though lesions in the orbit and pectoral muscles have been described [4, 8–11].

A retrospective series by Surov et al. reviewed 5170 patients with metastatic cancer, 61 of which were found to have skeletal muscle metastases. Of these, the iliopsoas muscle and paravertebral muscles were the most common sites of metastases at 27.5% and 25%, respectively. Interestingly, genital (24.6%) and gastrointestinal (21.3%) tumors were the most common primary sources while pulmonary malignancies accounted for only 0.8% [12]. Paravertebral involvement, as in the presented case, is thought to be of venous route via paravertebral venous plexus which dons connections to the inferior vena cava and mesenteric venous system [13].

An isolated, painful skeletal muscle metastasis as the initial manifestation of an otherwise asymptomatic primary malignancy, as in our patient, is uncommon [6, 9, 14]. Muscular metastases typically present as a painful and frequently palpable mass with or without localized swelling [2]. In 17 cases of skeletal muscle metastases of lung cancer reviewed by Di Giorgio et al., 7 of 17 had a metastatic mass as the sole manifestation of their neoplasm, with the mean age of diagnoses being 55.4 years. Eleven of these patients had concomitant nonmuscular distant metastases [15]. In the described case, a subsequent MRI revealed multiple additional intramuscular metastases—a phenomenon which has been described as exceedingly unusual—particularly in a relatively young patient of 39 years [16].

When a mass is seen on imaging, it is often difficult to distinguish between a primary sarcoma, a metastatic lesion, or an abscess as each can manifest with a broad spectrum of radiological features. It has been suggested that MRI is a superior modality for discovering muscle metastasis; however, masses can show identical characteristics on MRI or CT [3, 17]. Surov et al. described the most common CT findings of muscular metastases, from all origins, as a mass with homogenous enhancement. A subgroup analysis revealed that masses of pulmonary origin had low central attenuation with rim enhancement as the most common finding on CT [13]. Correlation with FDG-PET is recommended in all patients with a history of malignancy or a suspicion lesion [3, 8, 9, 18]. Biopsy of these masses is mandatory for proper diagnosis and further management [6, 9, 19].

The optimal strategy for addressing skeletal muscle metastases is unknown, but the presence of these lesions does not currently modify regimens of chemotherapy or radiation per current guidelines [15]. The presence of skeletal muscle metastases has been considered to herald a particularly aggressive tumor and linked to poor survival (often less than a year) by the time muscular metastases are diagnosed as the staging of the primary neoplasm is already advanced [2, 10, 15, 20, 21]. Pain, the most frequent complaint of these masses, is often drug resistant. In such cases, reports have described pain control and tumor reduction with palliative radiation [10, 15]. Wide excision of the mass, often coupled with radiation, has also been linked with pain control in addition to prolonged survival after muscle metastasis diagnosis [4, 19, 22].

## 4. Conclusion

Maintaining a high index of suspicion for malignancy when evaluating a persistent, painful soft tissue mass is essential in reaching a diagnosis early in the clinical course. In a review by Pop et al., 5-year survival in those patients whose skeletal muscle metastases were identified early enough to allow for surgical resection was nearly twice than that of those patients only treated with chemotherapy [23]. This case fuels the need for increasing awareness of muscular metastases as manifestations of a distant, lethal neoplasm.
